# The reality of rurality: Understanding the impact of remoteness on out‐of‐hospital cardiac arrest in Western Australia – A retrospective cohort study

**DOI:** 10.1111/ajr.13184

**Published:** 2024-09-10

**Authors:** Ashlea Smith, Stephen Ball, Karen Stewart, Judith Finn

**Affiliations:** ^1^ Prehospital, Resuscitation and Emergency Care Research Unit (PRECRU), Curtin School of Nursing Curtin University Bentley Western Australia Australia; ^2^ St John Western Australia Belmont Western Australia Australia; ^3^ Department of Epidemiology and Preventive Medicine Monash University Melbourne Victoria Australia; ^4^ Emergency Medicine, Medical School The University of Western Australia Perth Western Australia Australia

**Keywords:** emergency medical services, emergency medicine, epidemiology, rural health

## Abstract

**Introduction:**

Western Australia (WA) spans a large, sparsely‐populated area of Australia, presenting a challenge for the provision of Emergency Medical Service (EMS), particularly for time‐critical emergencies such as out‐of‐hospital cardiac arrest (OHCA).

**Objective:**

To assess the impact of rurality on the epidemiology, incidence and survival of OHCA in WA.

**Methods:**

We conducted a retrospective cohort study of EMS‐attended OHCA in WA from 2015 to 2022. Incidence was calculated on all OHCAs, but the study cohort for the multivariable regression analysis of rurality on survival outcomes consisted of OHCAs of medical aetiology with EMS resuscitation attempted. Rurality was categorised into four categories, derived from the Australian Standard Geographic Classification – Remoteness Areas.

**Results:**

The age‐standardised incidence of EMS‐attended OHCA per 100 000 population increased with increasing remoteness: Major Cities = 104.9, Inner Regional = 123.3, Outer Regional = 138.0 and Remote = 103.9. Compared to Major Cities, the adjusted odds for return of spontaneous circulation (ROSC) at hospital were lower in Inner Regional (aOR = 0.71, 95%CI 0.53–0.95), Outer Regional (aOR = 0.62, 95%CI 0.45–0.86) and Remote areas (aOR = 0.52, 95%CI 0.35–0.77) but there was no statistically significant difference for 30‐day survival. Relative to Major Cities, Regional and Remote areas had longer response times, shorter transport‐to‐hospital times, and higher rates of bystander CPR and automated external defibrillator use.

**Conclusions:**

Out‐of‐hospital cardiac arrest in rural areas had lower odds of ROSC at hospital compared to metropolitan areas, despite adjustment for known prognostic covariates. Despite WA's highly sparse regional population, these differences in ROSC are consistent with those reported in other international studies.

**What is already known about this subject?**

- Prehospital care is made more complex with increasing rurality, particularly for time critical emergencies such as out‐of‐hospital cardiac arrest.
- The odds of survival to 30 days are nearly 50% lower in rural areas compared to metropolitan in out‐of‐hospital cardiac arrest patients internationally.
- Emergency Medical Services response times are longer in rural areas compared to metropolitan areas.
- A variety of definitions and measurements of rurality are used internationally.

**What this study adds**

- A comprehensive epidemiological study of the impact of rurality on out‐of‐hospital cardiac arrest in Western Australia, with its unique, sparsely‐populated geography.
- Findings suggest that increasing rurality results in decreasing odds of return of spontaneous circulation on arrival at hospital.
- The community response to cardiac arrest is improved with increasing rurality; a higher proportion of CPR and automated external defibrillator use is noted in rural areas.
- These findings may help to inform ambulance and other health services to improve service dispersion and survival outcomes.

## 1 INTRODUCTION

Out‐of‐hospital cardiac arrest (OHCA) is a global health issue with typically poor 30‐day survival outcomes of less than 10%, even for emergency medical services (EMS)‐treated OHCAs.^1^ However, regional variation in OHCA survival has been well documented^2^, ^3^, ^4^ with increasing interest in the effect of rurality.^5^, ^6^ Analysing the impact of rurality is an important step in understanding the way population dispersion, patient demographics and healthcare system differences may impact time‐critical emergencies. A recent systematic review of 28 studies across 13 countries showed that, despite similar patient demographics and arrest characteristics, crude 30‐day survival rates from OHCA were approximately 50% lower in rural areas compared to metropolitan (metro) areas, and EMS response time was prolonged in rural areas.^7^ The review also found variable methods of defining rurality,^7^ with limited studies assessing a spectrum of increasing rurality in relation to OHCA in Australia.^8^, ^9^

Western Australia (WA) provides a unique opportunity to investigate the impact of rurality on OHCA. Occupying a land space four times the size of Texas, USA, and with an average population density of one person per five square kilometres,^10^ WA covers a large, sparsely‐populated geographical area of Australia. Nearly 80% of WA's population of 2.6 million is concentrated in the capital city, Perth, in the south‐west corner of the state.^10^ With the remaining 20% of the population spread across 2.5 million square kilometres, Western Australia represents an extreme example of rurality. We hypothesised that increasing levels of rurality in WA would be associated with increasingly poor OHCA survival outcomes, due to the increased challenges of delivering prehospital care in more remote areas. Therefore, this study aimed to compare patient demographics, arrest characteristics and prehospital management of OHCA across a spectrum of increasing rurality in WA, and to ascertain how rurality impacts OHCA incidence and survival outcomes.

## 2 METHODS

### 2.1 Study design and participants

A retrospective cohort study of all EMS‐attended OHCA cases in WA between 1 January 2015 and 31 December 2022 was conducted. Incidence rate (crude, age and sex‐specific and age‐standardised), patient (age, sex) and arrest characteristics (including bystander and EMS response) and survival outcomes (return of spontaneous circulation [ROSC] on arrival at hospital and 30‐day survival) were compared across four categories of rurality (described below) for all cases.

The study cohort for the multivariable regression analysis of rurality on survival outcomes comprised OHCA cases of medical aetiology^11^ who had an EMS‐attempted resuscitation (or any case that received an automated external defibrillator (AED) shock from a bystander), excluding EMS‐witnessed arrests.

### 2.2 Study setting

**FIGURE A1:**
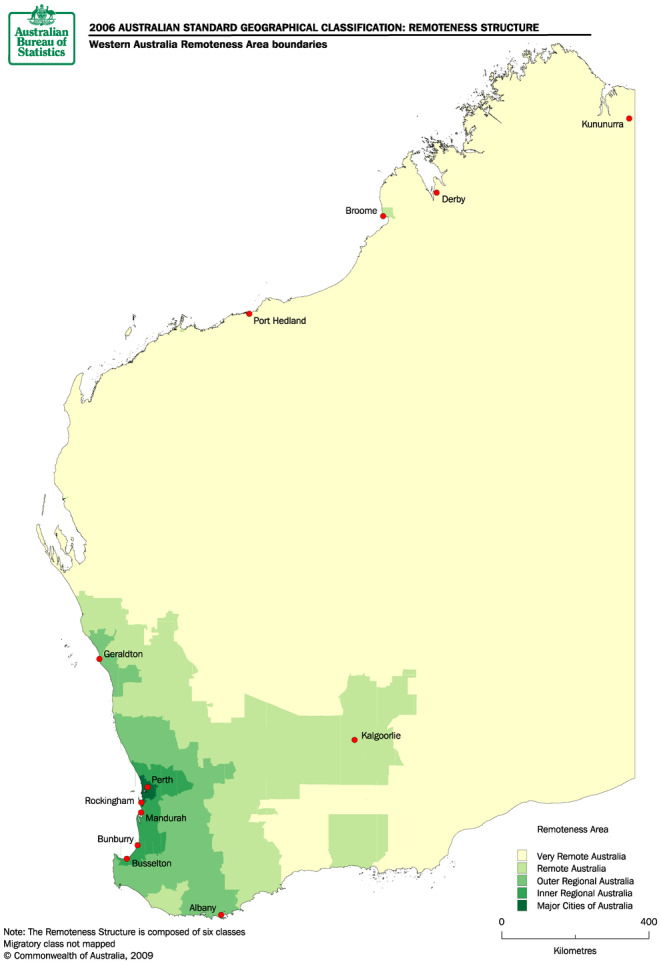
Western Australia Remoteness Area boundaries (*source*: https://www.ausstats.abs.gov.au/ausstats/subscriber.nsf/0/F894C5448550E411CA25796B0015028F/$File/1216015003_asgc_2006_ra_wa_map.pdf, cited 19/01/2021).

WA is divided into five rurality categories according to the Australian Standard Geographic Classification – Remoteness Areas (ASGC‐RA),^12^ as per Appendix: Table A1 and Figure A1 (methodology described in ‘Rurality Definition’ below). These categories, and their respective land area in WA, include Major Cities (2861 km^2^), Inner Regional (22 501.7 km^2^), Outer Regional (89 656.3 km^2^), Remote (167 348.9 km^2^) and Very Remote (2 244 264.4 km^2^).^13^ Within the Major Cities category is the only city, Perth, which covers 6400 km^2^ and has a population of 2.1 million people.^14^ Thus, Perth has a population density of 331 people/km^2^, compared to an average density of 0.2 people/km^2^ for the rest of WA, with the majority of the rural population residing in regional towns.^14^

The EMS for most of Western Australia is provided by St John WA (SJ‐WA). A standard response to OHCA in the metro area includes the dispatch of two ambulances, and an additional clinical support single vehicle, where available. Within rural WA, crew composition includes paramedic‐only crews (in the large regional town of Bunbury), mixed crews (i.e. registered paramedic and ambulance volunteer; present in larger towns), and volunteer‐only crews (in smaller towns/locations). Crews with a registered paramedic can perform advanced life support (ALS), including endotracheal intubation, vascular access and drug administration. Volunteer‐only crews perform basic life support (BLS), including defibrillation using an AED. There are approximately 150 EMS stations located across rural locations in WA.^15^ While SJ‐WA also provides helicopter EMS, and the Royal Flying Doctor Service^16^ also provides emergency response and fixed‐wing air retrieval for patients outside the metropolitan area, neither of these services provides a primary response to OHCA in WA. In the north‐west of the state, the rural towns of Derby, Fitzroy Crossing and Halls Creek and their vicinities, with an estimated population of 10 000 people, are serviced by the Kimberley Ambulance Service, provided by the Western Australia Country Health Service (WACHS).^17^ This area is not included in this study.

### 2.3 Data sources

The data for this study were sourced from the SJ‐WA OHCA Database, managed by the Prehospital, Resuscitation and Emergency Care Research Unit (PRECRU) at Curtin University, WA. This database comprises all EMS‐attended OHCA cases, collected from a combination of electronic patient care records (ePCR) as entered by paramedics, and computer‐aided dispatch data. The database variables align with the international Utstein OHCA definitions,^11^ and include patient age and sex, witness status (bystander‐witnessed, EMS‐witnessed, unwitnessed), bystander interventions (bystander cardiopulmonary resuscitation [B‐CPR], bystander AED shock delivery), initial arrest rhythm and time intervals including EMS response time, on‐scene time and time to hospital. For this study, initial arrest rhythm was coded as shockable/non‐shockable because volunteer ambulance officers only have access to AEDs. The capability of the attending EMS crew was also reported, where a crew was considered ALS capable if any advanced airway practice (i.e. intubation and supraglottic devices) and vascular access (i.e. intravenous and intraosseous) interventions were performed. The primary outcomes in this database are ROSC on arrival at hospital, and 30‐day survival, which is ascertained from the WA Death Registry^18^ for those patients not documented as deceased on the ePCR.

### 2.4 Rurality definition

The ASGC‐RA is a classification of increasing remoteness developed from the Accessibility/Remoteness Index of Australia (ARIA+).^12^ ARIA+ is a continuous measure of remoteness based on a calculation and indexing of road distance to the closest service centre^12^; defined as populated localities with available services, including health, education and retail resources.^19^ They are categorised according to the population size; localities with a population greater than 1000 people are considered to have a basic level of service.^20^ Five categories of rurality are denoted from this scale: Major Cities, Inner Regional, Outer Regional, Remote and Very Remote.^12^ For the purposes of this study, due to lower numbers of cases in both categories, Remote and Very Remote areas were combined to create four categories, henceforth referred to as: Major Cities, Inner Regional, Outer Regional and Remote. All OHCA cases had GPS coordinates, and cases were categorised according to their location and corresponding ASGC‐RA category.

### 2.5 Statistical analysis

Excel was used to calculate crude OHCA incidence, age‐ and sex‐specific incidence, and age‐standardised incidence using the Australian 2001 population as the standard.^21^

Patient and arrest characteristics, prehospital management and survival outcomes were compared between rurality categories using STATA.^22^ Survival outcomes of the Utstein comparator group (bystander‐witnessed, shockable arrests) were also compared. OHCA characteristics were reported as frequencies and percentages, or medians with interquartile ranges (IQRs). Comparisons were made using Chi‐squared tests for categorical variables and, depending on normal distribution, Kruskal–Wallis tests or *t*‐tests for continuous variables. *P*‐values less than 0.05 were statistically significant.

Multivariable logistic regression was conducted to identify the independent effect of rurality on the outcome variables of ROSC on arrival at hospital, and 30‐day survival. The exposure of interest (four levels of ASGC‐RA, with Major Cities as the reference category), was added to the model together with the Utstein covariates (defined a priori), namely age, sex, location (private residence/public place/residential care facility/other), witness status, initial rhythm, aetiology (presumed cardiac/other medical), bystander CPR, bystander AED shock delivered, ALS capability of attending EMS crew (ALS/BLS), EMS response time (from time of call answer until arrival at the scene), on‐scene time and transport to hospital time (from scene departure to hospital arrival), using the ‘Enter’ method, where all the input variables are entered simultaneously.^23^

## 3 RESULTS

### 3.1 Results for all OHCA cases

In total, there were 21 385 EMS‐attended OHCA cases during the study period (2015–2022): 15948 (74.5%) in Major Cities, 2142 (10.0%) in Inner Regional, 2022 (9.4%) in Outer Regional and 1273 (5.9%) in Remote areas. One Outer Regional case was excluded due to missing data on survival outcomes. The crude incidence of OHCA per 100 000 population was variable across the rurality spectrum (Major Cities = 104.9, Inner Regional = 123.3, Outer Regional = 138.0 and Remote = 103.9 – see Table 1). The age‐standardised incidence rate per 100 000 population increased across the rurality spectrum (Major Cities = 97.1, Inner Regional = 111.1, Outer Regional = 119.1 and Remote = 122.3). Age‐ and sex‐specific incidence rates are reported in Table S1.

**TABLE 1:** Characteristics of EMS attended OHCA in WA, stratified by rurality category.

Factor	Major cities	Inner regional	Outer regional	Remote	*p*‐Value
Total cases (*n*)	15 948	2142	2022	1273	
Population served (*n*)	1 916 849	217 039	183 185	83 603	
Geographic area (ha)	286 421.1	2 265 577.7	9 266 196.2	16 418 768.4	
Population density (persons/km^2^)	417.86	9.58	1.98	0.51	
Sex, male: *n* (%)	10 707 (67.1)^a^	1471 (68.6)	1422 (70.3)	907 (71.2)	0.008
Age, median (IQR)	66 (49, 79)	63 (46, 76)	64 (49, 77)	55 (39, 68)	<0.001
Adults (≥16 years)	15 691 (98.4)	2106 (98.3)	1989 (98.4)	1245 (97.8)	0.472
Incidence, per 100 000 population					
Crude	103.9	123.3	138.0	104.9	
Age‐standardized^b^	97.1	111.1	119.1	122.3	
Location					
Private residence	12 630 (79.2)	1502(70.1)	1464 (72.4)	808 (63.5)	<0.001
Public place	1898 (11.9)	473 (22.9)	370 (18.3)	355 (27.9)	
Residential care facility	1015 (6.3)	63 (2.9)	867 (4.3)	16 (1.2)	
Other	405 (2.5)	104 (4.8)	101 (5.0)	94 (7.4)	
Witness status, *n* (%)					
EMS	1135 (7.1)	127 (5.9)	99 (4.9)	61 (4.8)	<0.001
Bystander	23 776 (23.6)	523 (24.4)	447 (22.1)	312 (24.5)	
Unwitnessed	11 037 (69.2)	1492 (69.6)	1476 (73.0)	900 (70.7)	
Bystander CPR, *n* (%)					
Total	5860 (36.7)	815 (38.0)	716 (35.4)^c^	521 (40.9)^d^	<0.001
Bystander witnessed	2540 (67.2)	369 (70.5)	305 (68.2)	215 (68.9)^e^	0.007
Unwitnessed	3239 (29.3)	438 (29.3)	402 (27.2)	302 (33.5)	<0.001
Do not resuscitate order in place^f^, *n* (%)	1337 (8.4)	136 (6.3)	145 (7.1)	60 (4.7)	<0.001
Initial rhythm, *n* (%)					
Shockable (VT,VF,US)	1768 (11.1)	217 (10.1)	170 (8.4)	120 (10.2)	0.002
Non‐shockable (PEA^g^, asystole, bradycardia)	14 180 (88.9)	1925 (89.8)	1852 (91.6)	1143 (89.8)	
Bystander AED shock delivered for all cases, *n* (%)	287 (1.8)	47 (2.2)	26 (1.3)	36 (2.8)	0.008
Bystander AED shock delivered^h^, *n* (%)	247 (6.5)	36 (6.9)	15 (3.3)	30 (9.6)	0.006
Aetiology, *n* (%)					
Presumed cardiac	12 140 (76.1)	1434 (66.9)	1440 (71.2)	795 (62.4)	<0.001
Trauma	790 (4.9)	327 (15.2)	237 (11.7)	218 (17.1)	
Hanging	1189 (7.4)	181 (8.4)	149 (7.3)	151 (11.8)	
Drowning	143 (0.9)	26 (1.2)	26 (1.3)	31 (2.4)	
Drug overdose	698 (4.4)	46 (2.1)	40 (1.9)	15 (1.1)	
SUDI^i^	68 (0.4)	6 (0.3)	12 (0.6)	4 (0.3)	
Malignancy	565 (3.5)	76 (3.5)	77 (3.8)	46 (3.6)	
Anaphylaxis	17 (0.1)	2 (0.1)	1 (0.05)	1 (0.08)	
Other	10 (0.1)	0	0	1 (0.2)	
EMS resuscitation attempted, *n* (%)	7403 (46.4)	890 (41.4)	835 (41.3)	565 (44.4)	<0.001
EMS response time, median (IQR) (mins)	8.8 (6.7, 11.4)	12.8 (8.5, 20.2)	13.3 (8.7, 22.3)	15.3 (9.8, 26.6)	<0.001
Transport time to hospital, median (IQR) (mins)	10.1 (4.5, 13.7)	8.5 (5.0, 18.0)	6.1 (3.3, 11.4)	5.0 (3.0, 10.7)	<0.001
Died at scene: *n* (%)	11 549 (72.4)^j^	1678 (78.3)	1580 (78.1)^k^	909 (71.4)^l^	<0.001
ROSC (any): *n* (%)	2086 (13.1)	171 (7.9)	122 (6.0)	76 (5.9)	<0.001
ROSC (on hospital arrival): *n* (%)					
All cases	1657 (10.4)	134 (6.2)	95 (4.7)	63 (4.9)	<0.001
Utstein comparator group^m^	552 (46.8)	43 (30.7)	34 (33.0)	24 (28.2)	<0.001
Survival to 30 days: *n* (%)					
All cases	873 (5.4)	78 (3.6)	53 (2.6)	38 (2.9)	<0.001
Utstein comparator group^m^	429 (36.4)	32 (22.8)	23 (22.3)	21 (24.7)	<0.001

^a^ Excludes 2 cases with unknown sex.

^b^ Standardized using the Australian 2016 population.

^c^ 2 cases with unknown BCPR status.

^d^ 2 cases with unknown BCPR status.

^e^ 1 case of unknown BCPR status.

^f^ Do not resuscitate order per patient wishes in the form of a written advance care directive, verbal exchange with Medical Power of Attorney/family, or by registered medical doctor under which the patient is in care.

^g^ PEA, Pulseless Electrical Activity.

^h^ Of bystander witnessed cases‐ EMS witnessed excluded.

^i^ Sudden Unexplained Death in Infants.

^j^ 1 case with unknown status.

^k^ 2 cases with unknown status.

^l^ 1 case with unknown status.

^m^ Utstein comparator group‐ cases that were bystander witnessed with a shockable rhythm.

There were significant differences across the rurality categories for several patient and arrest factors (Table 1). OHCA cases in Remote areas had the lowest median age compared to other rurality categories (55 years, interquartile range 39–68, *p* = <0.001). Major Cities had the lowest proportion of arrests of traumatic aetiology (4.9%) compared to Inner Regional (15.2%), Outer Regional (11.7%) and Remote areas (17.1%), *p* = <0.001. Arrests in public places were variable across rurality categories, but highest in Remote areas (Major Cities = 11.9%, Inner Regional = 22.9%, Outer Regional = 18.3%, Remote = 27.9%, *p* = <0.001). Shockable rhythms had small but statistically significant differences across all rurality categories: (Major Cities = 11.1%, Inner Regional = 10.2%, Outer Regional = 8.4%, Remote = 10.2%, *p* = 0.002). The rates of bystander CPR were higher in Inner Regional (38.0%) and Remote areas (40.9%) when compared to Major Cities (36.7%), *p* = <0.001. Bystander AED shock delivered was low across all categories, but highest in Remote areas (Major Cities = 1.8%, Inner Regional = 2.2%, Outer Regional = 1.3%, Remote = 2.8%, *p* = 0.008). Increasing remoteness was associated with increasing median EMS response times (Major Cities = 8.8 min, Inner Regional = 12.8 min, Outer Regional = 13.3 min and Remote = 15.3 min, *p* = <0.001), but shorter median transport to hospital times (Major Cities – 10.1 min, Inner Regional – 8.5 min, Outer Regional – 6.1 min, Remote – 5.0 min, *p* = <0.001).

For all OHCAs, there was generally lower survival with increasing remoteness. Major Cities had the highest rate of ROSC on arrival at hospital (10.4%), with lower rates in Inner Regional (6.2%), Outer Regional (4.7%) and Remote (4.9%), *p* = <0.001. The Utstein comparator group had the highest rate of ROSC on arrival at hospital in Major Cities (46.8%) compared with all other groups (Inner Regional = 30.7%, Outer Regional = 33.0%, Remote = 28.2%, *p* = <0.001). Thirty‐day survival was highest in Major Cities (5.4%) compared with 3.6% in Inner Regional, 2.6% in Outer Regional and 2.9% in Remote areas (*p* = <0.001). Thirty‐day survival in the Utstein comparator group was highest in Major Cities (36.4%), with similar rates in Inner Regional (22.8%) and Outer Regional (22.3%), and Remote areas with the second highest rate (24.7%), *p* = <0.001.

### 3.2 Sub‐study cohort

**FIGURE 1:**
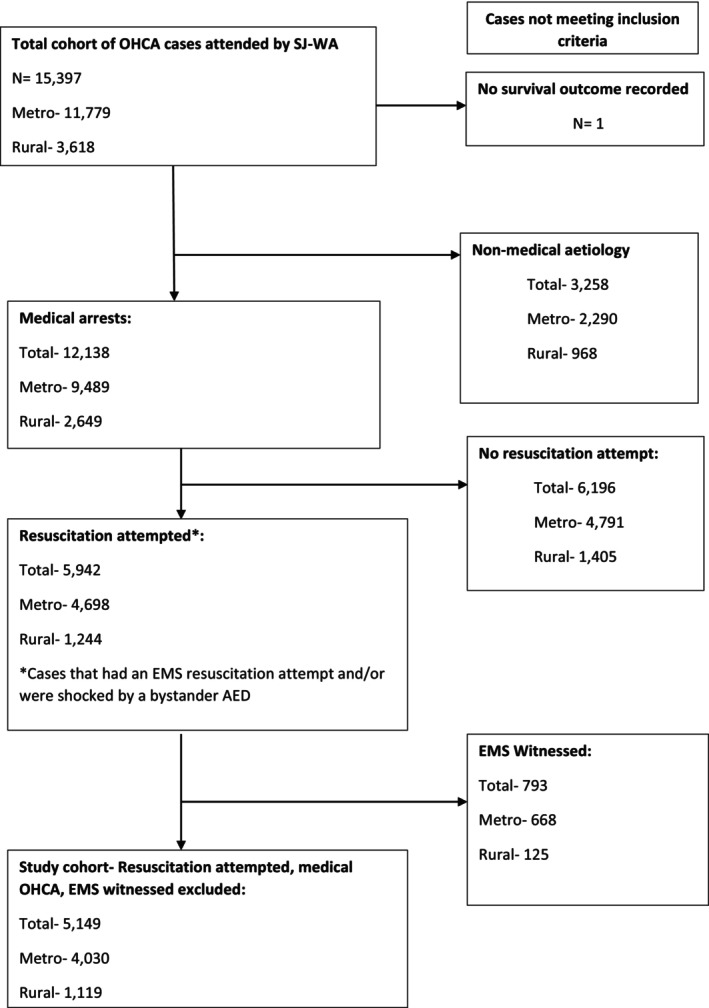
Sample selection flow chart.

After exclusion criteria were applied (Figure 1), the sub‐study cohort (medical aetiology and EMS‐attempted resuscitation, with EMS‐witnessed cases excluded) comprised 6866 OHCA cases: 5272 (76.7%) in Major Cities; 613 (8.9%) in Inner Regional; 603 (8.7%) in Outer Regional and 378 (5.5%) in Remote areas. Table 2 shows the differences in patient and arrest characteristics and survival outcomes between the four rurality categories for the study cohort, as reported below.

**TABLE 2:** Comparison by rurality category for all resuscitation‐attempted OHCAs of medical aetiology.

Factor	Major cities	Inner regional	Outer regional	Remote	*p*‐Value
Total cases, (*n*)	5272	613	603	378	
Sex, male, *n* (%)	3595 (68.2)^a^	433 (70.6)	408 (67.6)	257 (68.0)	0.637
Age, median (IQR)	68 (54, 79)	67 (53, 77)	65 (53, 77)	58 (47, 70)	<0.001
Adults (≥16yrs), *n* (%)	5145 (97.6)	597 (97.4)	591 (98.0)	372 (98.4)	0.668
Location, *n* (%)					
Private residence	3981 (75.5)	461 (75.2)	439 (72.8)	261 (69.0)	<0.001
Public place	771 (14.6)	97 (15.8)	99 (16.4)	71 (18.8)	
Residential care facility	373 (7.1)	25 (4.1)	32 (5.3)	10 (2.6)	
Other (e.g., prisons, medical centres)	147 (2.8)	30 (4.9)	33 (5.4)	36 (9.5)	
Witness status, *n* (%)					
Bystander	2786 (52.8)	323 (52.7)	298 (49.4)	194 (51.3)	0.431
Unwitnessed	2486 (47.1)	290 (47.3)	305 (50.6)	184 (48.7)	
Bystander CPR, *n* (%)					
Total	3849 (73.0)	492 (80.2)	462 (76.6)	295 (78.0)	<0.001
Bystander witnessed	2149 (77.1)	272 (84.2)	241 (80.8)	161 (83.0)	0.006
Unwitnessed	1700 (68.4)	220 (75.8)	221 (72.4)	134 (72.8)	0.027
Do not resuscitate order in place^b^, *n* (%)	359 (6.8)	28 (4.6)	37 (6.1)	12 (3.2)	0.009
Initial rhythm, *n* (%)					
Shockable (VT, VF, US)^c^	1411 (26.7)	175 (28.5)	138 (22.9)	109 (28.8)	0.092^d^
Non‐shockable (PEA^e^, asystole, bradycardia)	3861 (73.2)	438 (71.4)	465 (77.1)	269 (71.1)	
Bystander AED shock delivered^f^, *n* (%)	270 (5.1)	43 (7.0)	25 (4.1)	29 (7.6)	0.022
Aetiology, *n* (%)					
Presumed cardiac	4995 (94.5)	590 (96.2)	576 (95.5)	369 (97.6)	<0.001
Malignancy/palliative	146 (2.7)	12 (1.9)	11 (1.8)	5 (1.3)	
Respiratory	61 (1.1)	4 (0.6)	7 (1.1)	1 (0.2)	
SUDI^g^	56 (1.0)	6 (0.9)	8 (1.3)	2 (0.5)	
Anaphylaxis	14 (0.2)	1 (0.1)	1 (0.1)	1 (0.2)	
Cases where crew is ALS capable^h^, *n* (%)	5272 (100)	530 (86.4)	437 (72.4)	249 (65.8)	<0.001
EMS response time, median (IQR) (mins)	8.9 (6.9, 11.4)	12.5 (8.7, 18.9)	12.8 (8.8, 20.1)	14.0 (9.6, 20.0)	<0.001
Transport time to hospital, median (IQR) (mins)	10.0 (6.9, 13.3)	7.8 (4.9, 15.3)	5.7 (3.0, 10.0)	5.0 (3.0, 9.0)	<0.001
Died at scene, *n* (%)	2439 (46.2)^i^	315 (51.4)	305 (50.6)	151 (39.9)	0.011
ROSC (any), *n* (%)	1355 (25.7)	101 (16.5)	79 (13.1)	46 (12.1)	<0.001
ROSC (on hospital arrival), *n* (%)					
All cases	1053 (19.9)	81 (13.2)	62 (10.3)	38 (10.0)	<0.001
Utstein comparator group^j^	541 (47.4)	41 (31.5)	34 (35.0)	22 (28.5)	<0.001
Survival to 30 days, *n* (%)					
All cases	537 (10.2)	51 (8.3)	32 (5.3)	24 (6.3)	<0.001
Utstein comparator group^j^	422 (37.0)	31 (23.8)	23 (23.7)	19 (24.7)	<0.001

^a^ Excludes 2 cases with unspecified sex.

^b^ Do Not Resuscitate order per patient wishes in the form of a written Advance Care Directive, verbal exchange with Medical Power of Attorney/family or via registered medical doctor under which the patient is in care.

^c^ VT, Ventricular Tachycardia; VF, Ventricular Fibrillation; US, Unspecified Shockable.

^d^ P‐value for Chi Squared test of shockable vs non‐shockable.

^e^ PEA, Pulseless Electrical Activity.

^f^ Of bystander witnessed cases‐ EMS witnessed excluded.

^g^ Sudden Unexplained Death in Infants.

^h^ ALS skills include advanced airway skills (i.e., intubation, supraglottic devices) and vascular access (i.e., intravenous or intraosseous).

^i^ One case unknown regarding death at scene.

^j^ Utstein comparator group‐ cases that were bystander witnessed with a shockable rhythm.

#### 3.2.1 Patient and arrest characteristics

In the sub‐study cohort, there were small but significant differences between rurality categories in percentage of patients who were males (Major Cities = 68.2%, Inner Regional = 70.6%, Outer Regional = 67.6%, Remote = 68.0%, *p* = <0.001). There were no significant differences in the percentage of cases with an age greater than 16 years between the rurality categories (Major Cities = 97.6%, Inner Regional = 97.4%, Outer Regional = 98.0%, Remote = 98.4%, *p* = 0.668). The median age of the cases decreased with increasing remoteness (Major Cities = 68 years, Inner Regional = 67 years, Outer Regional = 65 years, Remote = 55 years, *p* = <0.001). Major Cities had the lowest proportion of OHCAs in public places (14.6%), with an increasing proportion in Inner Regional (15.8%), Outer Regional (16.4%) and Remote areas (18.8%), *p* = <0.001. The proportion of bystander‐witnessed OHCAs was similar across all categories (Major Cities = 52.8%, Inner Regional = 52.7%, Outer Regional = 49.4%, Remote = 51.3%, *p* = 0.431). The highest rates of bystander CPR were in Inner Regional and Remote areas, in both bystander‐witnessed (84.2% and 83.0% respectively, *p* = 0.006) and unwitnessed arrests (75.8% and 72.8% respectively, *p* = 0.027). Bystander‐administered AED shocks were highest in Inner Regional (7.0%) and Remote areas (7.6%) and lowest in Major Cities (5.1%) and Outer Regional areas (4.1%), *p* = 0.022.

#### 3.2.2 EMS crew capability and response time

All Major Cities cases were attended by ALS capable EMS crews, with increasing remoteness having reduced rates of ALS capable crews attending OHCA cases (Major Cities = 100.0%, Inner Regional = 86.4%, Outer Regional = 72.4%, Remote = 66.0%, *p* = <0.001). There was a trend towards longer median EMS response times with increasing remoteness: Major Cities = 8.9 min (IQR 6.9, 11.4), Inner Regional = 12.5 mins (IQR 8.7, 18.9), Outer Regional = 12.8 min (IQR 8.8, 20.1), Remote = 14.0 min (9.6, 20.0), *p* = <0.001. Conversely, median transport time to hospital decreased with increasing remoteness: Major Cities = 10.0 min (IQR 6.9, 13.3), Inner Regional = 7.8 min (IQR 4.9, 15.3), Outer Regional = 5.7 min (IQR 3.0, 10.0), Remote = 5.0 min (3.0, 9.0), *p* = <0.001.

#### 3.2.3 Unadjusted survival

Increasing remoteness was associated with decreased ROSC on arrival at hospital, with Remote areas having nearly half the rate of ROSC on arrival at hospital compared to Major Cities (10.0% vs. 19.9%, *p* = <0.001). The Utstein comparator group had the highest percentage of ROSC on arrival at hospital in Major Cities (47.4%) compared to all other categories (Inner Regional = 31.5%, Outer Regional = 35.0%, Remote = 28.5%, *p* = <0.001). Compared with Major Cities, the crude odds of ROSC on arrival at hospital were 39% lower in Inner Regional areas (OR 0.61, 95% CI 0.48–0.78); 54% lower in Outer Regional areas (OR 0.46, 0.35–0.60); and 56% lower in Remote areas (OR 0.44, 0.32–0.63) (Table 3).

**TABLE 3:** Multivariable analyses of resuscitation‐attempted, medical aetiology OHCA.

Factor	ROSC on arrival at hospital		30‐day Survival	
	Unadjusted OR (95% CI)	Adjusted OR (95% CI)^a^	Unadjusted OR (95% CI)	Adjusted OR (95% CI)^b^
Rurality				
Major cities	Ref.			
Inner regional	0.61 (0.48–0.78)	0.71 (0.53–0.95)	0.80 (0.59–1.08)^c^	0.96 (0.65–1.41)^c^
Outer regional	0.46 (0.35–0.60)	0.62 (0.45–0.86)	0.49 (0.34–0.71)	0.66 (0.42–1.04)^c^
Remote	0.44 (0.32–0.63)	0.52 (0.35–0.77)	0.59 (0.39–0.91)	0.68 (0.40–1.14)^c^
Age (effect per additional year of age)	0.99 (0.98–0.99)	0.99 (0.99–1.00)^c^	0.98 (0.97–0.98)	0.97 (0.97–0.98)
Sex				
Female	Ref.			
Male	1.12 (0.98–1.28)^c^	0.72 (0.61–0.85)	2.01 (1.64–2.46)	1.12 (0.88–1.44)^c^
Location				
Private residence	Ref.			
Residential care facility/Nursing home	0.60 (0.43–0.83)	1.19 (0.79–1.79)^c^	0.18 (0.07–0.45)	0.59 (0.23–1.56)^c^
Public place	3.26 (2.81–3.79)	1.15 (0.95–1.40)	6.44 (5.39–7.70)	1.50 (1.18‐ 1.91)
Other (e.g., prisons, medical centres)	2.32 (1.74–3.10)	1.54 (1.05–2.27)	3.21 (2.25–4.58)	1.48 (0.92‐ 2.38)^c^
Witness status				
Unwitnessed	Ref.			
Bystander witnessed	3.92 (3.39–4.53)	1.72 (1.45–2.04)	6.32 (5.04–7.94)	2.39 (1.83–3.12)
Aetiology				
Presumed cardiac	Ref.			
Other medical	0.43 (0.29–0.63)	0.62 (0.39–0.97)	0.25 (0.13–0.49)	0.40 (0.18–0.88)
Initial rhythm				
Non‐shockable	Ref.			
Shockable	6.02 (5.28–6.87)	1.59 (1.35–1.88)	23.14 (18.40–29.10)	5.91 (4.54–7.70)
Bystander CPR				
No	Ref.			
Yes	1.90 (1.62‐ 2.23)	1.25 (1.04, 1.52)	4.06 (3.06–5.39)	1.53 (1.11–2.11)
Bystander AED				
No	Ref.			
Yes	7.15 (5.75–8.88)	2.48 (1.86, 3.31)	12.68 (10.12–15.89)	2.21 (1.63–2.99)
ALS capability				
BLS	Ref.			
ALS	4.90 (3.00–8.01)	2.33 (1.32–4.12)	2.27 (1.39–3.72)	0.97 (0.51–1.87)
Response time (effect per additional minute)	0.93 (0.92–0.95)	0.94 (0.93–0.96)	0.93 (0.91–0.95)	0.92 (0.90–0.95)
Scene time (effect per additional minute)	0.95 (0.94–0.95)	0.99 (0.98–1.00)^c^	0.93 (0.93–0.94)	0.96 (0.95‐ 0.97)
Transport time (effect per additional minute)	1.01 (1.01–1.02)	1.02 (1.01–1.03)	1.01 (1.01–1.02)	1.02 (1.01–1.03)

^a^ Model *p*‐value ≤ 0.001.

^b^ Model *p*‐value ≤ 0.001.

^c^ NS, Statistically non‐significant.

Thirty‐day survival was lowest in Outer Regional areas (5.3%) compared with Major Cities (10.2%), Inner Regional (8.3%) and Remote areas (6.3%), *p* = <0.001. The Utstein comparator group (for cases of medical aetiology as per the sub‐study cohort) had the highest 30‐day survival in Major Cities (37.0%), with Remote areas having the second highest rate (24.7%) compared to Inner Regional (23.8%) and Outer Regional (23.7%), *p* = <0.001. While the crude odds of 30‐day survival were not statistically significant for Inner Regional areas (compared to Major Cities), the odds of 30‐day survival were lower in Outer Regional (OR 0.49, 0.34–0.71) and Remote areas (OR 0.59, 0.39–0.91).

#### 3.2.4 Multivariable logistic regression modelling

Increasing remoteness was associated with lower adjusted odds of ROSC on arrival at hospital, but no significant difference in the odds of 30‐day survival, relative to Major Cities (Table 3).

#### 3.2.5 ROSC on arrival at hospital

After adjustment for Utstein covariates and ALS capability, the odds of ROSC on arrival at hospital in all rurality categories were still lower compared to Major Cities, with lower odds with increasing remoteness (Inner Regional aOR 0.67, 0.51–0.90; Outer Regional aOR 0.56, 0.41–0.77; Remote aOR 0.43, 0.29–0.64). Factors significantly associated with improved odds of ROSC on arrival at hospital included bystanders witnessing the arrest (aOR 1.73, 1.46–2.05), a shockable initial rhythm (aOR 1.61, 1.36–1.89), bystander CPR (aOR 1.25, 1.03–1.51), bystander AED shock delivered (aOR 2.44, 1.83–3.25), and if the EMS crew had ALS skills capability (aOR 2.33, 1.32–4.12). The odds of ROSC on arrival at hospital were reduced as a function of EMS response time (aOR 0.94, 0.93–0.96, scaled as the per‐minute effect).

#### 3.2.6 30‐day survival

After adjustment, the odds of 30‐day survival in all rurality categories, relative to Major Cities, were lower but not statistically significant (Inner Regional aOR 0.97, 0.66–1.42; Outer Regional aOR 0.67, 0.43–1.04; Remote aOR 0.66, 0.40–1.10). The factors that had a statistically significant association with improved 30‐day survival in the multivariable model included: arrests in public locations (aOR 1.50, 1.17–1.91), a shockable initial rhythm (aOR 5.91, 4.54–7.69), bystander‐witnessed (aOR 2.39, 1.83–3.12), bystander CPR performed (aOR 1.53, 1.11–2.11) and bystander AED shock delivered (aOR 2.19, 1.62–2.98). The adjusted odds of 30‐day survival were reduced for EMS response time (aOR 0.92, 0.90–0.95; per‐minute).

## 4 DISCUSSION

Our comparison of OHCA across WA has highlighted the impact that increasing remoteness has on OHCA incidence, survival and EMS response. We found that there is a higher incidence of OHCA in Inner Regional and Outer regional areas when compared to Major Cities and Remote areas. Patients who experience OHCA in WA have lower crude odds of survival when in regional or remote areas than those in Major Cities areas. After adjusting for known prognostic covariates, the odds of ROSC on arrival at hospital were lower with increasing remoteness when compared to Major Cities by approximately 50%; however, there was no statistically significant difference in the odds of 30‐day survival. We observed differences in prehospital factors between the rurality categories. We expected to find that there were longer ambulance response times in rural areas. However, we were surprised that there were higher rates of bystander CPR and bystander AED usage in rural areas. Interestingly, similar rates of shockable rhythms were noted across all rurality categories, despite prolonged response times with increasing remoteness. Despite similar patient and arrest characteristics, and higher rates of bystander CPR, we noted a marked difference in survival rates. The differences in survival outcomes we observed are in line with international studies that had less extreme differences in average population density between metro and rural areas.^7^ Our findings suggest that there are unexplained differences in survival with increasing rurality in WA.

An unexpected finding from our study was the variation in bystander interventions across the rurality categories. Surprisingly, Remote areas had the highest rate of bystander CPR and AED shocks delivered of all categories. In Outer Regional areas, we noted lower rates of bystander‐witnessed arrests, bystander CPR and AED shocks delivered, and shockable initial rhythms compared to all other categories. These findings suggest that, while ARIA is a well‐recognised and utilised measure of the rurality spectrum across Australia, it does not necessarily account for fine‐scale population dispersion that may influence bystander presence relative to the location of cardiac arrests. While regional WA has a low average population density, the population dispersion and clustering patterns are not uniform across the regional area. Populations in WA tend to congregate within regional towns, with these towns varying in size and location; these are necessary factors for determining health service distribution.^24^ The observed short median transport times to hospital indicate that the arrests in regional areas may not be occurring very far from towns, where there is closer proximity to EMS and other health services, as well as a higher likelihood of bystander intervention. The exploration of population and service dispersion may provide more context to the observed difference between rurality categories and their respective bystander engagement rates, and therefore survival rates, in WA.

While WA has a comparably higher rate of bystander CPR to similar studies internationally,^7^ the rates of bystander interventions were highest in Remote areas of WA. Bystander interventions have been shown to improve survival outcomes in OHCA patients, particularly the provision of CPR.^25^, ^26^ Giotra et al.^2^ found that bystander CPR played an important role in the improvement in survival outcomes in OHCA patients, which we noted in the higher crude odds of both ROSC at hospital and 30‐day survival, when bystander CPR was performed. The higher rate of provision of both bystander CPR and AED use in WA may be not only due to increased community engagement but also due to closer relationships within smaller communities. In WA, St John WA facilitates community engagement in ambulance volunteer programs with clinical educators and paramedics, which may also foster the importance of the shared responsibility of the community and ambulance service in the early intervention for OHCA to ensure the best outcomes for patients.

There was a decline in the proportion of OHCA cases attended by ALS‐capable EMS crews as remoteness increased, and increased adjusted odds of ROSC on arrival at hospital if the attending crews had the capacity to perform ALS skills. This was also found in a study by Peters et al.,^6^ who also noted that rural OHCAs are more likely to be attended by BLS‐only EMS crew and that ALS care was associated with increased odds of ROSC.^6^ A systematic review and meta‐analysis by Bakalos et al.^27^ found a strong effect of higher odds of survival to 30 days when ALS care is given in OHCAs of medical aetiology. Further, the exposure of paramedics to cases requiring advanced skills, and the reduced access to training courses and equipment in rural areas were factors that Betts et al.^28^ highlighted as a disparity between metro and rural prehospital care. While our study only examined the effect of the capacity for ALS skills to be performed, the reduced availability of ALS‐capable crews, and the reduced opportunity for skills training and exposure of paramedics in rural areas may also be contributing factors in poorer survival outcomes.

A notable discrepancy across the rurality categories was the increasing ambulance response time in rural areas, a potentially modifiable factor for OHCA. The greater degree of variability in response time in rural areas is demonstrated in the wide interquartile range, of which Major Cities areas do not experience. We also found that the odds of survival decreased as a function of increasing ambulance response time. Ambulance response times internationally are consistently longer in rural areas than metro.^7^ While the response times in this study were similar to international averages, response times in rural areas were surprisingly reasonable despite the vast area covered by the ambulance service and overall low average population density. Previous studies have shown that shorter ambulance response times and early access to advanced life support result in increased rates of ROSC, survival to hospital discharge and survival with a favourable neurological status.^29^, ^30^, ^31^ An interesting finding from our study was that while rural areas had longer response times compared to Major Cities, these areas had shorter transport times to hospital. However, while the transport time to a hospital is shorter for rural areas, not all of these receiving facilities have 24 h access, and all of WA's public access cardiac catheterisation laboratories and intensive care units are located in metro Perth.^32^ Moreover, St John of God Bunbury Hospital is the only interventional cardiology capable hospital in rural WA; but it does not provide 24‐hour emergency access and is restricted to patients with private health cover. While the service provision and capability of the receiving hospitals does not influence the rates of ROSC at ED, this may have an impact on 30‐day survival, and further research is required to determine the actual impact.

Despite this delay in ambulance response in rural areas, similar rates of shockable rhythms were noted when compared to Major Cities areas; this may be due to the higher rates of bystander CPR. While short response times are the target, provision of ambulance services to rural areas is challenging, particularly within WA with sparsely populated areas surrounding larger rural towns. Therefore, rural communities need to maintain higher rates of bystander CPR and AED use to compensate for the delay in ambulance care. Initiatives such as education campaigns to train rural communities in basic life support may encourage bystander intervention and improve survival outcomes. A community‐based study conducted by Moller et al.^33^ aimed to evaluate the impact of a BLS education campaign delivered to a rural community, which found that the bystander intervention rate and OHCA survival rate both improved significantly. This study^33^ highlights the effectiveness and importance of educational campaigns, such as the recent Heart Matters trial,^34^ in OHCA survival improvement. While an engaged community is necessary to capitalise on the OHCA Chain of Survival,^35^ Masterson et al.^36^ also note that this does not mitigate a prolonged ambulance response, and improvement of survival requires a community‐specific assessment to ensure equitable and sustainable care for patients who experience OHCA in rural areas.

### 4.1 Limitations

Due to the lack of a standardised definition or scale of rurality internationally, defining rurality in a consistent manner with other similar studies is challenging.^7^ The scale used in our study is an accepted tool within Australian rurality classification, however, contains a wide variation in population density within its categories. Therefore, the assessment of population density alongside these categories might provide a more comprehensive understanding of the location and available services of the arrests.

Our study was unable to assess factors outside of prehospital care, including in‐hospital management of OHCA or provision of heart health‐related community initiatives and the effect these factors have, particularly on 30‐day survival. Factors such as patient comorbidities and tertiary care capabilities across WA, which could impact on 30‐day survival (in particular), were also not examined.

## 5 CONCLUSION

Out‐of‐hospital cardiac arrest patients in rural WA have lower crude survival than in metropolitan areas. After adjusting for patient and arrest characteristics, and EMS response time, the adjusted odds of ROSC at hospital remained lower in rural areas, but there was no statistically significant difference in 30‐day survival. Despite the extreme differences in average population density across WA, the poorer observed survival rates in rural and remote areas were consistent with international literature. Future research is required to explore additional factors (e.g. distance to tertiary care and population clustering) that may help explain differences in OHCA survival across the rurality spectrum of WA.

## AUTHOR CONTRIBUTIONS

**Ashlea Smith:** Conceptualization; investigation; writing – original draft; methodology; validation; visualization; formal analysis; project administration. **Stephen Ball:** Conceptualization; methodology; writing – review and editing; data curation; supervision. **Karen Stewart:** Data curation; supervision; writing – review and editing. **Judith Finn:** Conceptualization; funding acquisition; writing – review and editing; project administration; data curation; supervision.

## FUNDING INFORMATION

AS funded by Curtin University PhD Scholarship; JF funded by NHMRC Investigator grant GTN1174838.

## CONFLICT OF INTEREST STATEMENT

AS and KS are employees of SJ‐WA; SB and JF hold adjunct research positions with SJ‐WA and JF receives research funding from SJ‐WA.

## ETHICS STATEMENT

Ethical approval was granted from the Curtin Human Research Ethics Committee (Approval no. HR128/2013), as a sub‐study to the Western Australia Prehospital Care Record Linkage Project. Approval was also granted by the St John WA Research Governance Committee.

## Supporting information


Table S1.

## Data Availability

Data sharing is not applicable to this article as no new data were created or analyzed in this study.

## Appendix

**TABLE A1:** Structure of ASGC remoteness areas.

Class	Abbreviation	Index value range
Major cities of Australia	MC	0–0.2^a^
Inner Regional Australia	IR	>0.2–2.4^b^
Outer Regional Australia	OR	>2.4‐5.92^c^
Remote Australia	R	>5.92–10.53^d^
Very Remote Australia	VR	>10.53–15^e^
Migratory^f^		

*Source*: ABS (2001b).

^a^ Equal to or greater than 0 but less than or equal to 0.2.

^b^ Greater than 0.2 but less than or equal to 2.4.

^c^ Greater than 2.4 but less than or equal to 5.92.

^d^ Greater than 5.92 but less than or equal to 10.53.

^e^ Greater than 10.53 but less than or equal to 15.

^f^ Areas composed of off‐shore, shipping and migratory CDs. In allocating an ASGC Remoteness Areas class to an area of land, only the first five classes are applicable.
