# Supplementary material for: The reality of rurality: Understanding the impact of remoteness on out‐of‐hospital cardiac arrest in Western Australia – A retrospective cohort study
Source: Aust J Rural Health. 2024 Sep 10;32(6):1159–72. doi: 10.1111/ajr.13184 (PMC11640207; doi:10.1111/ajr.13184)
Supplement: Supplementary file 1 — Table S1. [file AJR-32-1159-s001.docx]

| **Incidence** | **Major Cities** | | **Inner Regional** | | **Outer Regional** | | **Remote** | |
| --- | --- | --- | --- | --- | --- | --- | --- | --- |
| **Age (years)** | **Male** | **Female** | **Male** | **Female** | **Male** | **Female** | **Male** | **Female** |
| 0-4 | 18.46295 | 15.15052 | 12.62808 | 9.865825 | 23.24598 | 15.37245 | 16.90046 | 6.927766 |
| 5-9 | 3.696843 | 2.462326 | 3.231644 | 6.924249 | 9.606517 | 0 | 4.367575 | 4.598124 |
| 10-14 | 4.442226 | 4.688588 | 11.74024 | 5.144739 | 6.146533 | 8.538251 | 8.153947 | 14.44085 |
| 15-19 | 24.99256 | 9.49307 | 81.14541 | 23.57749 | 49.68032 | 21.43674 | 62.73111 | 19.119 |
| 20-24 | 47.64315 | 16.11525 | 121.1667 | 35.54743 | 83.97801 | 38.50711 | 96.49316 | 24.96256 |
| 25-29 | 58.91275 | 18.71182 | 130.9122 | 44.17742 | 122.7378 | 19.39488 | 55.95787 | 54.26615 |
| 30-34 | 68.47319 | 20.18395 | 114.0338 | 45.42014 | 109.8901 | 44.46025 | 87.70869 | 56.53462 |
| 35-39 | 93.39791 | 33.40381 | 123.242 | 29.69121 | 129.0138 | 66.4528 | 80.96311 | 71.03618 |
| 40-44 | 99.45888 | 39.7409 | 123.5445 | 46.11938 | 130.0973 | 59.52381 | 137.7361 | 61.7284 |
| 45-49 | 134.7445 | 56.25056 | 159.8304 | 62.35191 | 157.0985 | 52.10746 | 129.7383 | 62.05482 |
| 50-54 | 150.2434 | 54.48382 | 141.9172 | 70.2982 | 205.7459 | 86.85917 | 165.4325 | 70.13379 |
| 55-59 | 197.3428 | 67.12437 | 167.4481 | 72.08766 | 230.2778 | 102.2061 | 205.8615 | 79.19103 |
| 60-64 | 245.9631 | 94.71922 | 255.4324 | 84.19318 | 270.6773 | 99.02242 | 279.38 | 92.95183 |
| 65-69 | 283.0456 | 104.2385 | 257.809 | 106.5726 | 363.5396 | 114.2996 | 318.5262 | 253.6574 |
| 70-74 | 431.8594 | 179.987 | 419.445 | 171.9278 | 440.5149 | 162.5911 | 544.9972 | 196.8504 |
| 75-79 | 554.3666 | 246.1288 | 567.6405 | 235.1218 | 536.0281 | 210.9705 | 592.4171 | 485.5716 |
| 80-84 | 765.7596 | 368.4986 | 672.189 | 378.4455 | 801.7289 | 421.4897 | 884.5438 | 288.1206 |
| 85-89 | 1089.714 | 541.6761 | 1012.5 | 584.3682 | 1091.174 | 454.5455 | 1048.658 | 372.0238 |
| 90-94 | 1588.72 | 825.0825 | 1170.799 | 915.109 | 1632.047 | 709.9828 | 1562.5 | 581.3953 |
| 95-99 | 2349.769 | 1182.246 | 2008.929 | 1041.667 | 1358.696 | 1196.809 | 357.1429 | 337.8378 |
| >100 | 2419.355 | 1679.104 | 0 | 0 | 5000 | 2205.882 | 0 | 0 |
| Total | 141.1074 | 67.62646 | 168.1773 | 77.87692 | 191.9922 | 82.84181 | 134.4918 | 67.9632 |

Supplementary Table 1- Age and sex-specific incidence of OHCA per 100,000 population
